# The Social Context of HIV Prevention and Care among Black Men Who Have Sex with Men in Three U.S. Cities: The Neighborhoods and Networks (N2) Cohort Study

**DOI:** 10.3390/ijerph16111922

**Published:** 2019-05-30

**Authors:** Dustin T. Duncan, DeMarc A. Hickson, William C. Goedel, Denton Callander, Brandon Brooks, Yen-Tyng Chen, Hillary Hanson, Rebecca Eavou, Aditya S. Khanna, Basile Chaix, Seann D. Regan, Darrell P. Wheeler, Kenneth H. Mayer, Steven A. Safren, Sandra Carr Melvin, Cordarian Draper, Veronica Magee-Jackson, Russell Brewer, John A. Schneider

**Affiliations:** 1 NYU Spatial Epidemiology Lab, Department of Population Health, NYU School of Medicine, New York, NY 10016, USA; william_goedel@brown.edu (W.C.G.); denton.callander@nyulangone.org (D.C.); Brandon.Brooks@nyulangone.org (B.B.); seann.regan@gmail.com (S.D.R.); 2 Center for Research, Evaluation, and Environmental & Policy Change, My Brother’s Keeper, Inc., Jackson, MS 39202, USA; dhickson@uhupil.org (D.A.H.); smelvin@oahcc.org (S.C.M.); cdraper@mbk-inc.org (C.D.); 3 Us Helping Us, People Into Living, Inc., Washington, DC 20010, USA; 4 Chicago Center for HIV Elimination, University of Chicago, Chicago, IL 60637, USA; ychen22@medicine.bsd.uchicago.edu (Y.-T.C.); reavou@medicine.bsd.uchicago.edu (R.E.); akhanna@medicine.bsd.uchicago.edu (A.S.K.); rbrewer@medicine.bsd.uchicago.edu (R.B.); jschnei1@medicine.bsd.uchicago.edu (J.A.S.); 5 Department of Medicine, University of Chicago, Chicago, IL 60637, USA; 6 Survey Lab, University of Chicago, Chicago, IL 60637, USA; hansonhd@uchicago.edu; 7 Pierre-Louis Institute of Epidemiology Public Health (UMR-S 1136), Faculté de Médecine Saint-Antoine, Sorbonne Universités, 75012 Paris, France; basile.chaix@iplesp.upmc.fr; 8 Iona College, New Rochelle, New York, NY 10801, USA; dwheeler@iona.edu; 9 The Fenway Institute, Fenway Health, Boston, MA 02215, USA; KMayer@fenwayhealth.org (K.H.M.); ssafren@miami.edu (S.A.S.); 10 Division of Infectious Diseases, Department of Medicine, Beth Israel Deaconess Medical Center, Harvard Medical School, Boston, MA 02215, USA; 11 Department of Psychology, College of Arts and Sciences, University of Miami, Coral Gables, FL 33124, USA; 12 Brotherhood Inc., New Orleans, LA 70119, USA; vmagee@brotherhoodinc.org; 13 Department of Public Health Sciences, University of Chicago, Chicago, IL 60637, USA

**Keywords:** HIV prevention, HIV care, geography, neighborhoods, networks, gay men’s health, men who have sex with men (MSM), minority, black, African American

## Abstract

*Background:* In many parts of the world, stark racial disparities in human immunodeficiency virus (HIV) prevalence, incidence, prevention, and care outcomes persist among gay, bisexual, and other men who have sex with men (MSM), with Black MSM significantly impacted in the United States (U.S.). Individual-level characteristics, including sexual behaviors and socioeconomic status, do not fully account for racial/ethnic disparities in HIV among MSM. We hypothesize that neighborhood contexts and network characteristics influence risk for HIV infection as well as HIV-related prevention and care behaviors. As such, the study design includes the use of real-time geospatial methods and in-depth assessments of multiple network typologies to investigate the impact of neighborhood and network-level factors on HIV prevention and treatment among Black MSM residing in longstanding priority HIV elimination areas in the U.S., namely Chicago, Illinois and in the Deep South (Jackson, Mississippi and New Orleans, Louisiana) (*n* = 450, *n* = 50, and *n* = 100, respectively). We describe the design, sampling methods, data collection, data management methods, and preliminary findings of the ongoing ‘Neighborhoods and Networks (N2) Cohort Study’. *Methods/Design:* N2 employs a prospective longitudinal design. The sample includes Black MSM participants in Chicago recruited via respondent-driven sampling and assessed every six months over two years of follow-up. Participants enrolled in Jackson and New Orleans are being recruited through existing health and community services and assessed every six months over one year of follow-up. Mobility within and between neighborhoods is being assessed using global positioning system (GPS) technology. Social and sexual networks among Black MSM are being studied through egocentric network inventories as well as newer methods of creating meso-level networks that involve social media (Facebook) and mobile phone contacts. Key HIV prevention outcomes such as pre-exposure prophylaxis (PrEP) care engagement, and HIV/STI (sexually transmitted infections) biomarkers will be examined at baseline and follow-up. *Results:* As of 31 December 2018, a total of 361 men were enrolled across all study sites: 259 in Chicago and 102 in the Deep South (75 in New Orleans and 27 in Jackson). At baseline, participants ranged in age from 17 to 65 years old (mean = 34.3, standard deviation = 5.1) with 123 men (34.1%) self-reported as HIV positive. While HIV treatment levels were similar between sites, men in the Deep South reported higher rates of adherence than men in Chicago (63.3% versus 49.4%, *p* = 0.03). Sexual risk profiles were mainly the same between men from different study sites, with 22.9% of men in Chicago and 28.9% in the Deep South reporting consistent condom use during vaginal and anal sex (*p* = 0.26). Regarding their home neighborhoods, men in the Deep South were more likely than those in Chicago to characterize theirs as having a good reputation (43.1% versus 24.7%, *p* < 0.001) and as being safe (37.3% versus 21.2%, *p* = 0.002). *Conclusions:* The focus on Black MSM in the N2 Study will allow for a nuanced exploration of the attitudes, beliefs, behaviors, and practices of a diverse group of Black MSM. The study is also positioned to provide novel insight about neighborhood and network characteristics that influence HIV-related behaviors. A health equity framework ensures that Black MSM are not explicitly or implicitly deemed as deviant, disordered, or the non-reference group. Findings from N2 will provide guidance for the implementation of more impactful HIV prevention interventions that engage a diverse population of Black MSM as we work toward HIV elimination in the U.S.

## 1. Background

Gay, bisexual, and other men who have sex with men (MSM) in the United States (U.S.) have been disproportionately impacted by human immunodeficiency virus (HIV) since the beginning of the epidemic in the early 1980s [1,2,3]. In 2015, there were 39,513 newly diagnosed cases of HIV infection and 66.8% were among MSM [2]. Between 2008–2014, the yearly incidence of HIV infection in the U.S. and a majority of transmission risk groups (e.g., people who inject drugs, high-risk heterosexuals) decreased, while the incidence rate among MSM overall remained stable [4]. If current trends in the prevention and care of HIV continue, it is estimated that one in six MSM will be diagnosed with an HIV infection in their lifetime [5].

Across the U.S., there is spatial and racial/ethnic heterogeneity in the prevalence and incidence of HIV among MSM and other at-risk groups. This heterogeneity relates to specific subgroups of MSM, such as Black/African American MSM (hereafter referred to as “Black MSM”), and also particular geographic locations in the U.S., including urban areas such as Chicago, Illinois [6] and cities such as Jackson, Mississippi and New Orleans, Louisiana in the Delta region of the Deep South [7,8,9,10], which has been typically defined to include Georgia, Alabama, Mississippi, and Louisiana. In these regions, Black MSM between 20–29 years old have the highest HIV rates of any subgroup [2]. Should current incidence rates persist, some estimates indicate that one in two Black MSM will be diagnosed with HIV infection within their lifetime [5].

In a landmark meta-analysis, Millett et al. (2012) examined racial disparities among key HIV care outcomes and found that Black MSM were less likely to access, utilize, and be adherent to antiretroviral treatment (ART) and thus less likely to achieve viral load suppression compared to MSM with other racial/ethnic backgrounds [11]. Racial disparities with respect to HIV care have been well established [12], and have been extended to effective HIV biomedical prevention such as pre-exposure prophylaxis (PrEP) [13]. These observations are the foundation for scientific movement beyond disparities research to improve rates of viral load suppression that drive onwards transmission within this diverse population.

A modified version of Urie Bronfenbrenner’s ‘social ecological model of human development’ suggests that individual-level biological and behavioral factors associated with HIV transmission and acquisition are influenced by increasingly broad layers of social and contextual factors, including neighborhoods and networks [14]. For example, neighborhood-level factors can shape access to and the utilization of safe and competent prevention and care services, promoting health and well-being or reinforcing various forms of stigma and discrimination [14]. Furthermore, social networks can provide support for individuals engaged in HIV care services and reinforce protective social norms to promote risk reduction behaviors [15]. In this model, HIV infection is not viewed as a consequence of individual behaviors, but it is acknowledged that there are social contexts where “… individuals lose their power to enact their protective intentions, or where unsafe practices are perhaps the only viable and adaptive survival strategy” [16].

As such, neighborhood contexts and network characteristics may influence one’s risk for HIV infection, as well as HIV-related prevention and care behaviors. There is a growing literature that has identified several salient neighborhood and network determinants of HIV-related outcomes among MSM [17,18,19,20]. First, physical characteristics of the built environment, such as the absence of public transportation infrastructures, may define and limit access to HIV prevention and care services [21,22]. For example, the presence of health-promoting institutions (e.g., lesbian, gay, bisexual and transgender (LGBT) community organizations, hospitals, clinics, LGBT associations, Pride events) within a geographic area as well as the distance between an individual’s home and these services have been associated with HIV testing uptake among MSM [23]. Second, in line with the minority stress model [24], social factors, such as homophobia, racism, and income inequalities at the neighborhood-level may further promote inequities in health by limiting access to care and exposing individuals to stigma and discrimination [25,26]. In addition, concentrations of poverty and other forms of social disadvantage can lead to social disorganization and the loss of social capital (i.e., features of social organization that improve efficiency of society by facilitating coordinated actions), in turn producing higher rates of crime and violence [27]. As a result, individuals exposed to these areas may be more prone to adopt maladaptive coping behaviors [28], such as engaging in substance use [29,30]. Moreover, living in these types of neighborhoods may lead to emotional trauma, which may undermine individual health preservation through the enactment of preventive behaviors.

MSM are also embedded in several types of networks (e.g., social and sexual networks) that may influence their risk of HIV infection. Previous research has shown that characteristics of sexual networks, such as age discordance (i.e., partnerships between individuals of different ages), concurrency (i.e., partnerships overlapping in time), and engaging in sex upon first acquaintance are significant risk factors for HIV and other sexually transmitted infections (STIs) [31,32]. Other research among MSM has demonstrated that the network viral load, or the amount of virus that surrounds given index clients, is associated with HIV serostatus [33]. There is some evidence suggesting that multiplexity (i.e., overlap) between social and sexual networks may be related to condomless anal intercourse and the transmission of HIV and other STIs among MSM [34]. This relationship is particularly relevant because other research has found HIV-related behaviors are more assortative (‘like with like’) within sexual compared with social networks among young Black MSM [35]. In a related analysis, Black MSM reporting a social network enabler (i.e., someone who would not disapprove of the respondent’s sexual risk behavior) were more likely to engage in condomless anal intercourse; this finding has not been observed in relation to their sexual network [35].

In addition, research has found that a greater family network proportion (i.e., having two or more family members in one’s close network) was associated with less frequent participation in group sex [36]. Other network studies have also found that specific structural positions within networks of young Black MSM were associated with HIV serostatus [37] and PrEP knowledge [38], and exhibit high degrees of homophily (i.e., persons are likely to form connections with others who share similar characteristics including socioeconomic status, values, beliefs, or attitudes) in terms of HIV status and sexual risk behaviors [35,39]. Further, young Black MSM exhibit a high turnover of their social networks [40], particularly around criminal justice involvement [41]. However, Black MSM also have organically generated social network groupings such as the house/ball community [42] (i.e., a subculture of LGBT Black and Latinx members that form collective safe spaces) [43] and “gay families” (i.e., alternative ‘chosen’ families that include traditional family roles such as mother and father) that can provide support and information around HIV prevention such as PrEP [44]. Finally, the rapid growth of mobile phone, social media, and “hook-up” apps allow for new kinds of analyses of virtual and digital networks [38]. Virtual and digital networks represent important archived network data and have been shown to provide important contextual information on risk at the individual or network level, as well as provide insights into predictions of sex positioning, HIV prevention communication potential, and the likelihood of diffusing novel interventions.

Naturally, neighborhoods and networks are closely related, and a growing body of work has explored interactions, including among MSM. One study found that a greater frequency of communication with social network members (defined as more than weekly) was associated with less concordance between one’s residential and social neighborhood among an urban sample of young MSM [45]. However, little work overall has been conducted on neighborhood and network determinants of HIV prevention in MSM, while the existing research has significant methodological limitations. Predominant among these is that most published studies on neighborhood-level determinants of health rely on crude static definitions of neighborhood areas: administrative boundaries such as ZIP codes and census tracts that are defined based on geography rather than the lived experiences of their residents [17]. The use of imprecise neighborhood definitions can result in spatial misclassification, or the incorrect characterization of a neighborhood-level exposure based on the neighborhood definition used [46,47,48].

Focusing on administrative boundaries also means that most of the existing research has focused disproportionately on residential neighborhoods [49,50]. However, the concept of ‘spatial polygamy’ suggests that people are exposed to multiple neighborhood environments throughout the course of their daily lives when working, socializing, and performing other aspects of life [51]. One study demonstrated that when residential neighborhoods defined using geographic information systems (GIS) were compared to “activity space neighborhoods” (those defined using global positioning system (GPS) devices), these two neighborhood definitions shared at most only 12% of the variance in the neighborhood characteristics studied [52]. This suggests that residential neighborhoods are a poor proxy for daily exposures to neighborhood characteristics, because most daily activities are conducted outside the context of these neighborhoods. Therefore, it is notable that GPS methods allow researchers to examine the multiple spatial contexts that an individual experiences (not just the residential neighborhood environment) and allow researchers to understand the timing and duration of exposures to specific spatial contexts (thereby solving the so-called ‘uncertain geographic context problem’) [49,53,54], both of which are significant advances in the field of HIV epidemiology and health promotion.

Emerging empirical research has shown that MSM overall, but especially Black MSM, participate in multiple neighborhoods throughout their daily lives, when working, attending school, socializing, and meeting sexual partners [45,55]. It has been argued that GPS methods are particularly relevant in measuring exposure to neighborhood environments [48,56], and research has shown these methods to be acceptable and feasible among MSM, including Black MSM [57,58]. The literature on neighborhoods and health among MSM also suffers from temporal ambiguity, as these studies rely on cross-sectional designs. Longitudinal designs extend the work of cross-sectional research by allowing the assessments of causal inference and can account for potential time lags between exposure to a neighborhood characteristic and change in a health behavior. Few studies on the neighborhood determinants of health, and no such studies with longitudinal neighborhood and network data, have been conducted among Black MSM.

Regarding studies assessing the role of networks on HIV prevention and care among MSM, a comprehensive understanding of the evolution of social and sexual networks in longitudinal cohorts of Black MSM is emerging [40]. Studies typically focus on either social networks or sexual networks, but there has been strong evidence suggesting a significant overlap between both network types among Black MSM [40,59]. There is also a need to extend the literature beyond analyses of local or personal networks to more accurately estimate structural network effects (e.g., bridging, centrality, link deletion) [37]. Studies frequently focus on egocentric networks and do not connect networks within the cohort by matching respondents’ networks to one another, so there has been little work assessing how various networks may connect and interact within a single population. Further, little research has assessed differences in the characteristics of networks that exist within and across neighborhoods [19], and very few studies have considered the possibility of synergistic effects of neighborhood and network characteristics, especially as they relate to HIV outcomes in MSM, including Black MSM [19]. Finally, very few studies have been conducted on neighborhood and network determinants of HIV prevention and care behaviors among Black MSM specifically.

**Figure 1:**
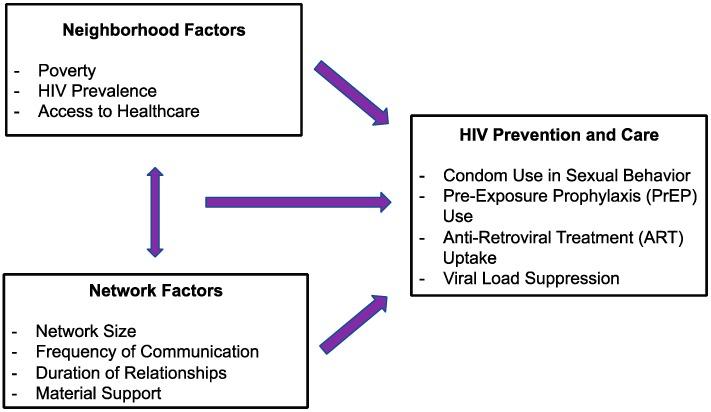
Conceptual framework for the influence of neighborhoods and networks on HIV prevention and care behaviors among Black men who have sex with men (MSM).

The overall objective of this study is to utilize real-time geospatial methods alongside in-depth assessments of multiple network typologies to investigate cross-sectional and longitudinal associations of neighborhood-level (e.g., poverty, HIV prevalence, and access to healthcare) and network-level (e.g., network size, frequency of communication with network members, and material support) factors on HIV prevention and care outcomes among Black MSM in areas that are home to large groups of this population in the U.S., namely the metropolitan statistical areas (MSAs) of Chicago, Illinois, Jackson, Mississippi, and New Orleans, Louisiana. In particular, the aim of this study is to examine the independent role of neighborhoods and networks in HIV outcomes, as well as the synergistic role of neighborhoods and networks in HIV outcomes (see Figure 1). This paper describes the design, sampling methods, and data collection and management methods of the ongoing Neighborhoods and Networks Cohort Study, which is also known as the “N2” Study.

## 2. Methods/Design

### 2.1. Overview of Study Design

The N2 Study employs a prospective longitudinal cohort design. All the sites started data collection in 2018: February 2018 in Chicago, August 2018 in New Orleans, and October 2018 in Jackson. The participants enrolled in Chicago are assessed every six months over two years of follow-up, for a total of five visits (one baseline assessment and four follow-up visits). Participants enrolled in Jackson and New Orleans are assessed every six months over one year of follow-up, for a total of three visits (one baseline assessment and two follow-up visits). The difference in length in the follow-up periods between sites is due to different funding mechanisms supporting each site. The study procedures described below are applicable to all sites.

During each visit, participants complete a digital survey instrument, which is an in-depth assessment of their social and sexual networks including digital networks and are provided with a GPS device to be worn prospectively for two weeks. At the completion of the assessment, participants receive compensation for their time and effort. At the end of the two-week GPS protocol, participants return the GPS device and receive compensation for their time and effort for carrying the GPS device and receive HIV/STI test results. At all sites, we split the survey into two sessions, which is in line with our incentive structure. Compensation for participation is based upon local standards with an average of $20 for each hour of study participation. In addition, participants who were eligible to receive $20/participant were referred as part of the respondent-driven sampling (RDS) process in Chicago and New Orleans.

At the baseline (and all follow-up) visits, all the participants received HIV testing if previously HIV negative. Indeed, participants who self-report their HIV status as negative or unknown are tested for HIV infection and provided with appropriate referral services as appropriate. At all the study sites, participants are tested for STIs at each visit, including syphilis and gonorrhea/chlamydia (multi-compartment).

Participants who test negative for HIV infection at their previous appointments continue to receive HIV testing with appropriate pre-test and post-test counseling at all sites. Should a participant test positive for HIV infection or infection with another STI during the follow-up period, they receive active linkage to care for appropriate medical and social support. Appendix A provides information on the research centers and study sites contributing to and facilitating N2.

### 2.2. Governance and Organizational Structure

The conduct of N2 is built around a community-based participatory model, which means that the study involves ongoing community member engagement to guide the design, development, and implementation. Each site periodically holds meetings of specially convened community advisory boards in order to support ongoing input and engagement. These meetings include reviewing study protocols and survey questions, receiving progress reports on the study, the presentation of data and their interpretation, and discussions around the implications of the study for Black MSM, including how the results of the study may inform HIV prevention and treatment priorities by community organizations and policymakers. In addition to the community advisory board, a scientific advisory board has been convened. The scientific advisors have been instrumental in informing the conceptual study design and the data analysis plan to analyze the data collected from the study. Periodically, the scientific advisory board convenes to review the progress of the study and provide guidance toward ensuring the collection of high-quality data.

### 2.3. Ethical Oversight

The N2 Study’s protocol has been reviewed and approved by several Institutional Review Boards (IRBs), namely: The New York University School of Medicine IRB (overall study; i16-01515 CR2 and i16-02158_CR2), The Biological Sciences Division/University of Chicago Medical Center IRB (Chicago site; IRB16-1419), and the Sterling IRB (Deep South sites; 5897 and 6304). Written consent was obtained from the study participants. Additionally, Certificates of Confidentiality from the National Institute of Health (for the Chicago site) and from the Center for Disease Control and Prevention (for the Jackson and New Orleans sites) have been obtained in order to protect the privacy of study participants against compulsory legal demands, such as court orders and subpoenas, as participants may report on potentially illegal behaviors.

### 2.4. Participant Recruitment

As of 31 December 2018, recruitment for N2 is ongoing with anticipated baseline completion date of August 2019. We aim to recruit a total 450 participants in Chicago, 100 in New Orleans, and 50 in Jackson. The recruitment strategies were flexibly designed to build upon existing research and service projects among Black MSM in these geographic locations. For the Chicago site, seed participants to initiate the RDS chain referral process [60] are drawn from a cluster of cohort, intervention, and service projects [38,40,44,61,62] as well as ongoing HIV testing research and service programs providing PrEP related care to young Black MSM [63]. As in previous work [40], ‘seed’ participants are invited to refer up to six members of their social networks, who then are also afforded the same opportunity. For the Jackson site, participants are recruited from an earlier cross-sectional study of GPS-defined neighborhood contexts associated with drug use and HIV infection among Black MSM, while New Orleans participants are recruited through existing service delivery and recruitment events, and is further supported by the distribution of printed advertisements at local colleges and universities, bars, clubs, and community-based organizations providing services to Black MSM, in-person recruitment from local bars and clubs frequented by Black MSM, direct messaging via social networking sites (e.g., Facebook, Twitter) and geosocial networking smartphone sex and dating applications (e.g., Grindr, Jack’d), and peer referral.

### 2.5. Inclusion and Exclusion Criteria

To be eligible for participation in the N2 Study in Chicago or the Deep South, participants must have: (a) identified as Black or African American; (b) been assigned male sex at birth and identify as male; (c) resided in the site MSA; (d) reported at least one sexual encounter with another man or a transgender women in the past year; (e) been willing to wear a GPS device; and (f) have no plans to move from the MSA during the course of the study. Chicago participants could be a minimum of 16 years of age, and we capped the age at 34 years of age at this site, which is in line with the highest HIV rates in this community, while in the Deep South, participants were aged 16 years or older, due to the limited knowledge about the networks and neighborhoods of Black MSM in these communities.

#### 2.5.1. Data Collection 1: Self-Report Survey Instruments

At the scheduled baseline (enrollment) and follow-up visits, participants complete a study questionnaire in private interview rooms to complete a study questionnaire. An illustrative list of survey measures contained in the study instruments is displayed in Table 1, which were selected in consultation with the N2 community and scientific advisory boards. They capture data on the understudied multi-level constructs needed to understand the influence of neighborhoods and networks on HIV prevention and care behaviors among Black MSM, including the potential mediators, confounders, or effect modifiers of any hypothesized associations. The survey is mixed mode in that some parts are self-administered, while others are administered by a trained interviewer. This approach to survey research can help decrease misclassification—particularly of sensitive questions (e.g., mental health) and those prone to socially desirable responses (e.g., drug use)—develop interviewer–participant rapport, and provide opportunities for participants to clarify questions about which they are uncertain [64].

#### 2.5.2. Data Collection 2: Neighborhoods Assessment

Mobility within and between neighborhoods is determined using GPS technology, which is a cutting-edge method to more accurately study neighborhood exposures [48]. To ensure the equivalent implementation of GPS protocols across study sites, key study staff (e.g., site principal investigators, project coordinator, and research assistants) participated in an interactive overview and training workshop to familiarize them with the GPS technology and protocol, which was conducted by members of New York University (NYU)’s Spatial Epidemiology Lab. Interview staff were trained to use the GPS units and taught the important points to discuss with participants (e.g., what the device can and cannot record).

At each survey assessment, participants were asked to wear and charge the GPS device over 14 [14] consecutive days. Participants were instructed to place the unit into their pockets or bags and keep the device with them at all times during the two-week period with the exception of when sleeping, swimming, or bathing. Participants were instructed to charge the device daily. In addition, participants received a travel diary containing two questions for each day of the protocol: “Did you charge the GPS unit today?” (Response Options: “Yes” and “No”) and “Did you carry the GPS unit with you today?” (Response Options: “Yes, for all journeys”, “Yes, for some journeys”, “No, but did make journeys”, and “I did not travel today”) [57,65]. Participants were sent reminder text messages to carry and charge the GPS device via text message during the protocol period. Example text messages were: “Phone, keys, wallet, GPS device!”; “New match on the dating app? Your GPS device wants to come too!”; and “Going out of town this weekend? Don’t forget to pack your GPS device and charger!”.

The GPS device itself was small (Qstarz BT-Q1000XT, manufactured by Qstarz International Co., Ltd., Taipei, Taiwan) and accompanied by two chargers (one for charging the device using a standard wall outlet and one car adapter for charging the device in a car). We considered using standard GPS apps within smartphones, but elected not to; this was mainly due to the development work yet required for this technology, which currently faces a number of challenges including severe battery drainage. This Qstarz device has been used in previous studies of neighborhoods and health [66,67], and has previously demonstrated acceptability and feasibility among MSM and other populations [57,58,68]. The device is programmed to log the participant’s current latitude and longitude coordinates at 10-second intervals, meaning that under ideal conditions, the device will record a latitude–longitude coordinate 360 times in the course of one hour, as done in previous studies [69,70,71]. This interval was selected as a compromise between optimal frequency (i.e., 1 s) and the device’s battery drain and data storage capacity over a two-week period. We decided to use the 10-s interval because our pilot study using varying recording intervals (i.e., 5 s, 10 s, 15 s, and 30 s (a 30-s logging interval is standard in the literature) found that the 10-s interval provides the best balance between battery and memory constraints and data quality. In addition, we implemented a 10-s GPS protocol in our pilot study of transgender women [72].

Upon completing the GPS protocol, participants returned to the study site to return their device and complete the travel diary. Data were imported from each device using QTravel, Qstarz’ proprietary software, and stored as GPX files labeled with the participant identification number and the device serial number. After exporting data, each GPS device’s log is cleared for use by future participants. Quality assurance on the imported GPS data was undertaken using Python software (version 2.7), scripts that remove errors (e.g., data with erroneous latitude and longitude values), and by using QGIS software (version 2.18), which facilitates a ‘nearest neighborhood analysis’ in order to remove the data points that suddenly move large distances (defined as more than 100 m in one move), as these likely reflect errors due to multipath reflectance [73].

In addition to defining neighborhoods with GPS data, we note that because we were collecting residential mailing addresses, we geocoded this information into residential neighborhoods. Data from the U.S. Census and American Community Survey [74,75] have been collected to approximate each component of the aforementioned theories and their influences on health outcomes. We will also obtain locally collected data, including access to relevant healthcare services, such as PrEP providers. In addition to these GIS measures of neighborhood characteristics, the study survey conducted prior to the two-week GPS period includes several items on perceived neighborhood characteristics, such as neighborhood safety, neighborhood stigma (i.e., places that are labeled socially and/or economically marginalized), neighborhood collective efficacy (i.e., social cohesion and informal social control), and gay neighborhood social norms (e.g., perceived neighborhood homophobia) [76].

#### 2.5.3. Data Collection 3: Social and Sexual Networks Assessment

Social and sexual networks were assessed during the study visit at which participants returned the GPS device. Egocentric social network analysis is an analytic approach that is used to understand the local structure, function, and composition of network ties around an individual (‘ego’), while sociocentric network analysis includes all of the individuals and ties connecting them within a boundary specification. In this study, we studied the social and sexual networks of MSM utilizing egocentric network inventories similar to those used in large national surveys, including the General Social Survey [77] and the National Social Life, Health, and Aging Project [78]. These inventories utilized a “name generator” question during the course of face-to-face interviews to elicit social and sexual network members who may influence the participant’s behaviors. These generators elicit names or other identifiers to be entered into a roster. Each participant was asked how many friends (‘confidants’) and sex partners with whom they have had sex in the past six months. This generator is then followed up with name interpreters, including a series of questions about each of the first five network members elicited (e.g., age, gender, race/ethnicity). As with previous social and sexual network studies with MSM [59,79], participants in the current study provided information about each network member, including demographics, their relationship with the participant, the network member’s health attitudes and behaviors, details on PrEP disclosure, PrEP encouragement and discouragement, and the extent to which network members were engaged with PrEP. In addition, from the social and sexual networks inventory generated from each participant, the network size and frequency of communication with network members were calculated as well as measures of emotional support, material support, and network turnover.

Multiple methods were used to create a larger structure to the egocentric networks described earlier—what we refer to as meso-level networks—to understand how the networks of multiple participants within the same population may overlap and connect. First, from the network inventory mentioned above, added structure was created using entity resolution approaches used in previous studies [37], including adaptive algorithms [80] to match named/extracted network members to both participants and the larger pool of network members elicited. Second, as completed in previous studies [38,81], data from Facebook and cell phone contact lists provide an additional method of adding structure to networks. Entity resolution is easily completed with digital data given the unique identifiers in the form of phone numbers or Facebook aliases. Facebook data were obtained from participants using an application within Facebook that allows the extraction of a ‘friends’ list (i.e., Facebook contacts) into a comma separated value (CSV) file. A freely available smartphone application was used to extract contact lists from participants’ cell phones to a CSV file. A dataset with information on the relationships between participants and between participants and their non-participating contacts could thereby be compiled from each data source. Finally, exploratory analyses will be conducted to examine how the multiplexity of networks was related to neighborhood activities and the prevention and PrEP-seeking behavior among Black MSM.

#### 2.5.4. HIV Prevention and Care Continuum Outcomes

For HIV-negative participants, the primary outcomes include self-reported prevention continuum variables related to PrEP. These variables include one’s awareness and use of PrEP, which will be assessed through a battery of validated self-report measures administered at baseline and follow-up visits [13]. If participants were on PrEP, self-reported adherence was also examined. Some other secondary behavioral measures include: willingness to use PrEP, PrEP discontinuation and rationale, barriers to PrEP uptake, and HIV testing. HIV-negative participants also received diagnostic testing for HIV to confirm their status and identify incident infections during the cohort’s follow-up period.

For HIV-positive participants in the cohort, the primary outcomes include self-reported linkage and retention in care, initiation of and adherence to ART, and viral load suppression, which will be assessed through validated measures at baseline and follow-up visits [82].

For both HIV-negative and HIV-positive participants, the secondary outcomes of the cohort include the history of STIs, the frequency of condomless insertive and/or receptive anal sex with a serodiscordant or unknown HIV-status partner, the frequency of alcohol and drug use in the context of sex, sexual partner numbers, and the number of group sex events. These outcomes will be measured with a battery of validated self-report measures administered at baseline and follow-up visits [82]. Additional information on clinical activities will be obtained.

#### 2.5.5. Covariates

To account for confounders that may explain observed associations, participants respond to survey items on their age, ethnicity, sexual orientation, socioeconomic status (education, income, occupation), current living situation, nativity, relationship status, drug use, depression, past incarceration status, health insurance status, disclosure of same-sex sexual behavior to primary care physician, residential self-selection, and residential history.

#### 2.5.6. Retention Plans

Participant retention is widely recognized as a vital feature of robust, reliable cohort research. The N2 Study retention plan is based on a multi-pronged approach designed to engage participants in the study and deliver participation reminders. First, at each visit study, the interviewers worked with participants to complete a contact information form, which gathered details on social media accounts (e.g., Facebook aliases) and the phone numbers for one friend and one family member who would be likely to know a participant’s whereabouts if direct contact failed. This process directly supports retention by increasing the likelihood of reaching participants for subsequent visits. Second, study staff engaged regularly with participants via birthday and holiday messages and the distribution of a study newsletter, which were strategies designed to help remind participants of their involvement in N2. Third, participants received a check-in call one month prior to each follow-up visit. Fourth, two follow-up reminders (calls, text messages, or social media messages) were delivered one week and one day ahead of a participant’s appointment. Fifth, in addition to free HIV and STI testing and financial incentives, retention was supported by the distribution of branded study paraphernalia (e.g., water bottles, backpacks).

Retention was also supported by this study’s partnerships with local organizations and services, noting that these organizations have developed decades-long relationships with community leaders and the general Black MSM community in each city. Through these relationships, N2 has been established as a community-based study with support from respected figures and organizations, which supports retention through the building of community-level rapport and trust. Further, the study’s partner organizations promoted the study while providing their programs and services, which provided a point of contact between study cycles. Finally, the majority of study interviewers were themselves members of the local Black MSM communities, which provides a further mechanism for decreasing attrition rates. With this plan, it is our goal to achieve a minimum of 80% retention of participants over the life of N2.

As this study employs a two-week period during which participants carry a GPS device, N2 will also build upon successes in earlier pilot work evaluating retention for GPS protocols. In a pilot cross-sectional study of MSM in New York City, all 75 participants returned their GPS device due, in part, to telephone and text messaging reminders issued at the end of the two-week period [57], which were results that were achieved in a similar pilot study of Black MSM in the Deep South [58].

### 2.6. Data Management, Quality Assurance and Control, and Statistical Analysis

As previously described, N2 collects four distinct data sources: (a) survey data, (b) GPS data, (c) digital network data, and (d) HIV and STI testing data. To link between these sources, each participant is assigned a unique numerical identifier that is consistent over the life of this study. As part of each visit, study interviewers reviewed participants’ files to ensure that each source of data had been collected and took steps to collect any missing pieces.

For the neighborhood analyses, we will apply standard and multi-level regression methods, while recognizing that there is potential for spatial autocorrelation in regression residuals—which can bias effect estimates and standard errors [83,84,85,86]. Therefore, if spatial autocorrelation is detected, we will implement spatial regression models [83,84,85,86]. We will use multiple analytic approaches for the network data. First, for egocentric network data, we will calculate proportions of network members with specific characteristics and examine how these and other local measures such as density, ego-betweeness, and others are related to core variables of interest. These egocentric network characteristic values could then be entered into standard statistical models. Following entity resolution as outlined above, we will explore use of several sociometric measures related to centrality and bridging. We will utilize tie and node estimation modeling approaches as appropriate and depending upon what structures are observed. Additionally, we will fit exponential random graph models (ERGMs), which provide a statistical framework for examining the structural properties of the network, the results of which complement our analysis of the egocentric networks [87,88]. In addition, we will work on integrated networks and neighborhood analyses. As appropriate, all analyses will be conducted among the full sample and were also site-specific, with the understanding of the potential for effect modification by geographic location. Further, as possible, we will take an intersectional analysis (recognizing heterogeneity in Black MSM), including examining effects by past incarceration status.

In addition, because we will have a cohort including HIV-negative participants, we will be also able to examine the incidence of HIV. Furthermore, we will be able to examine longitudinal trends in HIV prevention behaviors, including PrEP use and adherence, in the same cohort (as opposed to repeated cross-sectional samples). Lastly, we will be able to calculate incidence rates for bacterial STIs. For each of these outcomes, regression analyses will be employed to identify predictive factors, including those specifically related to neighborhoods and networks.

## 3. Findings to Date

As of 31 December 2018, a total of 361 men were enrolled across study sites, including 259 in Chicago and 102 in the Deep South (75 in New Orleans and 27 in Jackson). As of 31 December 2018, 29 participants had completed Wave 2 in Chicago with preliminary retention rate of 95%. No participants completed Wave 2 at the other study sites at that time. At baseline, participants ranged in age from 17 to 65 years old with an average of 34.3 (standard deviation = 5.1) (Table 2).

Table 2 provides an overview of the sociodemographic characteristics and some information on participant sexuality as reported at baseline. Although the majority of participants self-identified as gay or homosexual (56.0%), it is notable that nearly a third were bisexual (29.4%) and 24 men (6.6%) identified as straight or heterosexual; the remaining 29 (8.0%) participants chose other labels such as ‘queer’ or ‘pansexual’ or chose not to report their sexual orientation. Almost 70% (66.5%) reported a household income of <$25,000 and 105 participants (29.1%) reported some experiences of housing instability in the six months prior to participation.

Table 3 provides an overview of HIV and sexual health practices data, including self-reported baseline HIV prevalence. Overall, 123 men (34.1%) self-reported as HIV positive at enrollment, which did not differ between Chicago and the Deep South. However, among men with HIV, while treatment uptake was similar between sites, in a univariate analysis, we identified higher levels of self-reported ART adherence among men in the Deep South than in Chicago (63.3% versus 49.4% ‘always or almost always’ adherent, *p* = 0.03). Consistent condom use for anal or vaginal sex was low among our sample but similar between sites, with 22.9% of men in Chicago and 28.9% in the Deep South reporting always using a condom (*p* = 0.26). Participants from the Deep South were much more likely than those in Chicago to report having previously paid for sex (i.e., as a sex work client; 18.6% versus 3.9%, *p* < 0.001) but at the univariate level, there was no difference in the proportion who reported being paid for sex (16.9% of the overall sample were paid for sex or received some other compensation at least once in the 12 months prior to participation).

Table 4 reports on neighborhood characteristics at baseline. The features that men highlighted as important in choosing a home neighborhood were the same between those based in Chicago and those in the Deep South: being quiet, reasonably priced, and close to a downtown core were rated as the most important. By contrast, living around certain populations (Black people, White people, gay men) were ranked as the least important, suggesting that this kind of consideration does not commonly feature as a desirable aspect of these men’s home neighborhood. Overall, men rated the following problems as the most salient for their neighborhoods (in order): litter and trash, drug dealing, loiterers, police harassment, and abandoned houses. Each of these factors except for abandoned houses were more commonly reported by men in Chicago than those in the Deep South.

Table 5 reports on a selection of characteristics describing members of participants’ sexual and social networks at baseline. Men in Chicago named a total of 608 social network members (‘confidants’) and 587 sexual network members, while men in the Deep South named 328 confidants and 123 sexual partners. Overall, men individually named an average of 3.3 confidants and 2.5 sexual partners.

Overall, excluding network members for whom gender details were not provided, the majority of participants’ networks comprised cisgender men (57.2% of confidants and 74.0% of sexual partners), with men in the Deep South reporting a higher proportion of male confidants than those in Chicago (62.3% versus 54.4%, *p* = 0.022), but a lower proportion of men in their sexual networks (76.9% versus 89.1%, *p* = 0.002). Regarding HIV status, excluding those with no response, participants reported that 16.4% of their social networks and 16.1% of their sexual networks were HIV positive, with sizeable differences between study sites: 19.1% of confidants in Chicago were HIV positive compared to 8.5% in the Deep South (*p* < 0.001) as were 18.5% and 4.8%, respectively, of sexual partners (*p* = 0.002). It is notable that HIV status information was unknown or not reported for a much larger proportion of network members in the Deep South sites than those in Chicago.

As of 31 December 2018, GPS data had been collected for 306 participants (84.8% of the total sample). Collection of GPS data was higher in Chicago (91.5%) than in the Deep South (67.6%, *p* < 0.001). Across sites, the average number of days the GPS trackers were worn by participants was 10.1 (SD = 4.1).

## 4. Discussion

The N2 Study intends to collect rich and novel data from a sample of Black MSM in Chicago and the Deep South, reflecting the disproportionate rates of HIV born by this population and the special need for attention to these parts of the U.S. The primary analysis for this study will explore how these features of neighborhoods and networks affect the health and well-being of Black MSM, including their uptake of and adherence to PrEP. By doing so, the N2 study will facilitate a deeper and more nuanced understanding of HIV risk and prevention among Black MSM in areas with some of the highest rates of infection in the U.S.

Data collection is still underway. Based on our preliminary data of 361 men who are HIV-negative and HIV-positive enrolled across study sites, two-thirds of the sample reported a household income of <$25,000, and almost one-third reported some experiences of housing instability in the six months prior to participation. In our sample, consistent condom use for anal or vaginal sex was low, which is not surprising. Preliminary analyses also suggest that Black MSM form diverse social and sexual networks while reporting a high proportion of problems in their home neighborhoods. Also in terms of neighborhood characteristics, one interesting (perhaps unsurprising) finding was that 67% of the sample reported police harassment/abuse in their neighborhoods, and also 65% reported lack of police presence/response. In terms of networks, men individually named an average of 3.3 confidants and 2.5 sexual partners. Also, we found it interesting that HIV status information was unknown or not reported for a much larger proportion of network members in the Deep South sites than those in Chicago.

The aims of this research are in line with several of the goals enumerated in the National HIV/AIDS Strategy for the U.S. [89] as well as President Trump’s February 2019 State of the Union Address goal to end the HIV epidemic within 10 years in U.S. [90]. The focus on prevention-related behaviors and outcomes directly aligns with the goal of reducing new HIV infections, while the focus on care-related behaviors and outcomes directly aligns with the goal of increasing access to care and improving health outcomes for people living with HIV/AIDS [89]. In addition, by examining the role of multi-level factors that move beyond the individual on HIV infection and care outcomes, this cohort will contribute substantial empirical evidence to improving HIV-related health equity among MSM [89].

The traditional HIV care continuum assesses differences in stages of living with HIV from diagnosis to successful treatment [91], and many cohort studies conducted among MSM focus on either HIV prevention or treatment [92,93,94,95]; thus, the investigative team believes that an integrated care continuum that is status-neutral is essential to ending the HIV epidemic [91,96], which we aimed to do in this study. For people living with HIV, a positive test result is followed by high quality, culturally competent and sensitive care that will support individuals to engage/re-engage in care [97]. It is predicted that the empirical findings of this project will contribute to the development of polices that achieve the goals of the National HIV/AIDS Strategy for the U.S. [89] and reduce the burden of the HIV epidemic among Black MSM [2]. Through a comprehensive assessment of individual-level behaviors, attitudes, and beliefs while analyzing the influence of neighborhood-level and network-level factors, the current study aimed to elucidate both macro-level facilitators and barriers to improved health among this population through a coordinated HIV prevention and care response.

We fully recognize that cohort studies have been conducted among MSM to assess changes in risk for HIV infection over time. The Project 18 cohort [98] for example was developed to assess longitudinal determinants of syndemic production among a new generation of racially and ethnically diverse young MSM in New York City. Other cohorts have been created to assess differences between MSM of different racial/ethnic backgrounds over time. For example, the Involve(men)t cohort [94,99] was created to understand multi-level determinants of Black and White MSM in Atlanta, Georgia. However, few cohorts have been created to focus exclusively on the health of Black MSM without comparing them to MSM of other racial/ethnic backgrounds [100]. One such prospective cohort study focused exclusively on Black MSM is the HIV Prevention Trials Network (HPTN) 061 (BROTHERS), which included 1153 Black MSM followed for one year in six US cities: Atlanta, Boston, Los Angeles, New York City, San Francisco, and Washington, DC [100,101]. HPTN 061 is very different from our N2 cohort (beyond different regional focuses), as HPTN 061 was a feasibility study of a multi-component intervention to reduce HIV infection, while N2 focuses on the social contextual determinants of HIV. The exclusive focus of HPTN 061 and the N2 Study on Black MSM represents a next-generation health equity approach [102], allowing for rigorous intersectionality analyses such as a more nuanced understanding of the intra-group attitudes, beliefs, behaviors, and practices of Black MSM without explicitly or implicitly deeming them as deviant or disordered compared to White MSM [103]. Indeed, as compared to other racial/ethnic comparison-type studies and analyses, the study will measure the considerable diversity within Black MSM, allowing for within-group analyses of factors associated with resilience as well as the development of novel variables and constructs that are potentially related to prevention care continuums and well-being. These foci allow for the understanding that the experiences of Black MSM are not monolithic, allowing for an understanding of the varied behavioral risk profiles for individuals in these communities [103]. This, in turn, allows for the development of interventions that can be implemented among the most highly impacted individuals within these communities to directly influence the burdens and disparities experienced by Black MSM regarding HIV prevention and care in the U.S.

### Strengths and Limitations

The N2 Study is not without limitations. First, the cohort will be specific to Black MSM in three MSAs in the U.S. (one in the Midwest and two in the Deep South); therefore, our findings may not be generalizable to Black MSM in other geographic locations, such as Black MSM on the West Coast. Of note, this study was not designed to be representative of all Black MSM, but rather is intended to gain an understanding of the structural underpinnings that contribute to the increased prevalence, incidence, prevention, and management of HIV in these important geographical areas. Another limitation of this study is the use of different recruitment strategies between study sites, which may challenge comparisons between men in Chicago and the Deep South. Our decision to implement individually tailored approaches to recruitment was based on the feasibility expressed by our community partners, building on their unique understanding of the populations they serve and the methods that are most likely to reach men in different cities. To account for the potential biases between these groups, any comparative analyses will employ techniques of statistical matching on key sociodemographics to more accurately make comparisons across study sites.

This study also has a variety of strengths beyond the novel methodological approaches. The high prevalence of HIV infection among Black MSM in these three locations warrants the creation of the kind of large-scale sample that is expected via N2 in order to investigate the supra-individual factors associated with both HIV prevention and care behaviors and outcomes over time among these highly marginalized and vulnerable populations in order to inform the development of high-impact interventions and relevant policies. For example, increasing community efforts to combat LGBT hate crime rates within neighborhoods through increased local police attention in high-crime locations may serve as an intervention if an association between exposure to LGBT hate crimes and condomless anal intercourse is observed. Second, understanding the geographic navigation of Black MSM will also help inform where health services should be delivered. For example, given that we will know the travel patterns of Black MSM, we can identify high-traffic geographic locations for HIV testing/prevention/care interventions, which is an advancement of the literature, since such interventions are not often spatially targeted. In addition, targeting key network members can potentially facilitate the identification of Black MSM for HIV testing and linkage to care and enhancing social support, potentially increasing retention in HIV prevention and care services, such as the uptake of new biomedical interventions (e.g., PrEP) among Black MSM. Thus, the N2 Study provides an unprecedented opportunity to understand how multi-level factors can influence HIV outcomes among diverse Black MSM, which can have direct implications for planning behavioral and policy-level HIV prevention and care interventions.

## Figures and Tables

**Table 1:** Example Survey Constructs in the *Neighborhoods and Networks (N2) Cohort Study*.

Domain	Topics
Early Life Experiences	Adverse Childhood Experiences, Childhood Peer Victimization
Identity	Internalized Racism, Internalized Homophobia, Identity Congruence
Housing	Current Living Arrangement, Housing Affordability
Neighborhood Perceptions	Spatial Stigma, Social and Physical Disorder, Collective Efficacy
Mental Health	Depression, Anxiety, Post-Traumatic Stress Disorder
Substance Use	Tobacco Use, Alcohol Use, Illicit Drug Use
Sexual Behavior	Female Partners, Male Partners, Transgender Female Partners
HIV Care	Linkage to Care, Retention in Care, Viral Load
HIV Prevention	Perceived Risk for HIV Infection, Exposure to Prevention Activities
Healthcare	Health Literacy, Health Insurance Coverage, Medical Mistrust
Social Media and Technology	Smartphone Ownership, Social Media Use
Socioeconomic Circumstances	Financial Hardship, Relationship Status, Nativity

**Table 2:** Sociodemographic characteristics at baseline among cisgender Black gay, bisexual, and other men who have sex with men participating in the *Neighborhoods and Networks (N2) Cohort Study*, enrolled as of 31 December 2018.

	Total		Chicago		Deep South		
	*n*	%	*n*	%	*n*	%	*p*-diff ^a^
**Total participants**	361		259		102		
**Age range in years (M; SD)**	17–65 (34.3; 5.1)		17–36 (26.0; 4.1)		21–65 (37.5; 10.8)		0.04
**Highest education received**							
Some high school	54	15.0%	33	12.7%	21	20.6%	0.02
High school	128	35.5%	102	39.4%	26	25.5%	
Some secondary education	179	49.6%	124	47.9%	55	53.9%	
**Employment status**							
Employed or studying	185	51.2%	135	52.1%	50	49%	0.60
Not employed, not studying	176	48.8%	124	47.9%	52	51%	
**Annual income**							
<$25,000	240	66.5%	185	71.4%	55	53.9%	<0.001
≥$25,000	102	28.3%	73	28.2%	29	28.4%	
Not reported	19	5.3%	1	0.4%	18	17.6%	
**Housing** ^b^							
Homeless	105	29.1%	80	30.9%	25	24.5%	0.74
Live alone	77	21.3%	53	20.5%	24	23.5%	
Live with partner	22	6.1%	15	5.8%	7	6.9%	
Roommates	43	11.9%	32	12.4%	11	10.8%	
Others	114	31.6%	79	30.5%	35	34.3%	
**Sexual orientation**							
Gay	202	56.0%	147	56.8%	55	53.9%	0.46
Bisexual	106	29.4%	72	27.8%	34	33.3%	
Straight	24	6.6%	20	7.7%	4	3.9%	
Other or not reported	29	8%	20	7.7%	9	8.8%	
**Previous sexual partners** ^c^							
Cisgender men	337	93.4%	250	96.5%	87	85.3%	<0.001
Cisgender women	169	46.8%	132	51.0%	37	36.3%	0.01
Transgender women	72	19.9%	54	20.8%	18	17.6%	0.49
**Current relationship status**							
Not in a relationship	231	64.0%	163	62.9%	68	66.7%	0.41
Relationship with a man	106	29.4%	77	29.7%	29	28.4%	
Relationship with a woman ^d^	20	5.5%	17	6.6%	3	2.9%	
Relationship with multiple partners	4	1.1%	2	0.8%	2	2.0%	

^a^ Differences assessed using Chi-squared (categorical) or ANOVA (continuous); ^b^ In the previous 12 months; ^c^ Non-exclusive categories; ^d^ Includes cisgender and transgender women.

**Table 3:** HIV and sexual health practices at baseline among cisgender Black gay, bisexual, and other men who have sex with men participating in the *Neighborhoods and Networks (N2) Cohort Study*, enrolled as of 31 December 2018.

	Total		Chicago		Deep South		
	*n*	%	*n*	%	*n*	%	*p*-diff ^a^
**Total participants**	361		259		102		
**HIV status**							
Unknown	9	2.5%	7	2.7%	2	2.0%	0.82
HIV negative	229	63.4%	162	62.5%	67	65.7%	
HIV positive	123	34.1%	90	34.7%	33	32.4%	
**HIV testing** ^b,c^							
No test	52	21.8%	39	23.1%	13	18.8%	0.70
One or two times	54	22.7%	39	23.1%	15	21.7%	
Three or more times	132	55.5%	91	53.8%	41	59.4%	
**HIV pre-exposure prophylaxis** ^c^							
Never taken	150	63.0%	105	62.1%	45	65.2%	0.37
Previously taken	32	13.4%	26	15.4%	6	8.7%	
Currently taking	56	23.5%	38	22.5%	18	26.1%	
**HIV treatment** ^d^							
Never taken	5	4.1%	2	2.2%	3	9.1%	0.07
Previously taken	7	5.7%	7	7.8%	0	0.0%	
Currently taking	111	90.2%	81	90.0%	30	90.9%	
**HIV treatment adherence** ^e^							
Always/almost always take medication	59	48.0%	40	49.4%	19	63.3%	0.03
Sometimes forget to take medication	35	28.5%	31	38.3%	4	13.3%	
Often forget to take medication	17	13.8%	10	12.3%	7	23.3%	
**Recent condom use** ^f,g^							
Consistent condom use	81	24.5%	55	22.9%	26	28.9%	0.26
Inconsistent condom use	249	75.5%	185	77.1%	64	71.1%	
**Sex work or transactional sex** ^f^							
Paid someone for sex (client)	29	8.0%	10	3.9%	19	18.6%	<0.001
Recently paid for sex (worker)	61	16.9%	40	15.4%	21	20.6%	0.37
Previously paid for sex (worker)	33	9.1%	26	10.0%	7	6.9%	
Never paid for sex (worker)	267	74.0%	193	74.5%	74	72.5%	
**Group sex participation** ^f^							
No previous group sex	197	54.6%	140	54.1%	57	55.9%	0.68
Recent group sex	96	26.6%	72	27.8%	24	23.5%	
Previous groups sex	68	18.8%	47	18.1%	21	20.6%	
Age of first sexual experience (M; SD)	3–31 (14.9; 4.0)		3–26 (14.6; 3.6)		4–31 (15.6; 4.7)		0.02

^a^ Differences assessed using Chi-squared (categorical) or ANOVA (continuous); ^b^ In the previous 12 months; ^c^ Among HIV negative and unknown status participants; ^d^ Among HIV positive participants; ^e^ Among HIV positive participants on treatment; ^f^ ‘Recent’ defined as six months prior to participation, ‘previous’ defined as more than six months prior; ^g^ ‘Consistent’ condom use refers to always using condoms during anal or vaginal sex with partners of any gender, excluding participants with no recent anal or vaginal sex.

**Table 4:** Neighborhood characteristics at baseline among cisgender Black gay, bisexual, and other men who have sex with men participating in the *Neighborhoods and Networks (N2) Cohort Study*, enrolled as of 31 December 2018.

	Total		Chicago		Deep South		
	*n*	%	*n*	%	*n*	%	*p*-diff ^a^
**Total participants**	361		259		102		
**Connections in home neighborhood**							
No family, no friends	79	21.9%	55	21.2%	24	23.5%	0.21
Some family, no friends	33	9.1%	23	8.9%	10	9.8%	
No family, some friends	109	30.2%	72	27.8%	37	36.3%	
Some friends, some family	140	38.8%	109	42.1%	31	30.4%	
**Important neighborhood features** ^b^							
Reasonable cost	281	77.8%	202	78.0%	79	77.5%	0.91
Quiet	184	51.0%	130	50.2%	54	52.9%	0.64
Access to downtown area	146	40.4%	108	41.7%	38	37.3%	0.44
Near family and friends	109	30.2%	76	29.3%	33	32.4%	0.58
Mostly Black	52	14.4%	34	13.1%	18	17.6%	0.27
Mostly White	34	9.4%	24	9.3%	10	9.8%	0.88
Mostly gay	47	13.0%	32	12.4%	15	14.7%	0.55
**Current neighborhood problems** ^c^							
Litter, trash	263	72.9%	200	77.2%	63	61.8%	0.003
Drug dealing	252	69.8%	189	73.0%	63	61.8%	0.04
Adults and teenagers on the street	220	60.9%	173	66.8%	47	46.1%	0.001
Empty/abandoned houses	237	65.7%	177	68.3%	60	58.8%	0.09
Police harassment/abuse	241	66.8%	185	71.4%	56	54.9%	0.003
Lack of police presence/response	235	65.1%	176	68.0%	59	57.8%	0.07
Social group disagreements	218	60.4%	164	63.3%	54	52.9%	0.07
Graffiti	186	51.5%	143	55.2%	43	42.2%	0.02
**Neighborhood perceptions** ^d^							
Neighborhood has good reputation	108	29.9%	64	24.7%	44	43.1%	<0.001
Residents viewed negatively by others	151	41.8%	117	45.2%	34	33.3%	0.04
Neighborhood is safe	93	25.8%	55	21.2%	38	37.3%	0.00
**Recent negative events** ^e^							
Fight with a weapon	210	58.2%	166	64.1%	44	43.1%	<0.001
Someone was jumped or robbed	189	52.4%	162	62.5%	27	26.5%	0.007
Robbed, or property damaged	95	26.3%	69	26.6%	26	25.5%	0.82

^a^ Differences assessed using Chi-squared (categorial) or ANOVA (continuous); ^b,c^ Non-exclusive categories, ranked as ‘important’ or ‘very important’; ^d^ Non-exclusive categories, participants who ‘agree’ or ‘strongly agree’; ^e^ Non-exclusive categories, reported at least once in the six months prior to participation.

**Table 5:** Characteristics of sexual partners and social confidants at baseline among cisgender Black gay, bisexual, and other men who have sex with men participating in the *Neighborhoods and Networks (N2) Cohort Study*, enrolled as of 31 December 2018.

	Networks									
	Confidants (Social Network)					Sex Partners (Sexual Network)				
	Chicago		Deep South			Chicago		Deep South		
	*n*	%	*n*	%	*p*-diff ^a^	*n*	%	*n*	%	*p*-diff ^a^
	608		328			587		123		
**Age**										
<20 years old	20	3.3%	5	1.5%	<0.001	25	4.3%	1	0.8%	<0.001
20–29 years old	340	56%	141	42.9%		379	62%	53	40.1%	
≥30 years old	247	40.1%	180	54.9%		176	28.9%	69	56.1%	
Not known/refused/missing	1	0.2%	2	0.6%		7	12.4%	0	0.0%	
**Education**										
High school or less	273	44.9%	112	27.8%	0.19	273	46.5%	35	28.5%	0.006
Above high school	238	39.1%	120	29.8%		148	25.2%	38	30.9%	
Not known/refused/missing	97	16.0%	96	29.3%		166	28.3%	50	40.7%	
**Employment status**										
Employed full-time or part-time	428	71.3%	214	65.2%	0.027	375	63.9%	65	52.9%	0.002
Unemployed	166	27.7%	73	23.2%		149	25.4%	21	17.1%	
Retired	8	1.32%	12	3.7%		0	0.0%	2	1.6%	
Not known/refused/missing	6	1.0%	26	7.9%		63	10.7%	35	28.5%	
**Gender**										
Cisgender man	331	54.4%	200	61.0%	0.022	523	89.1%	93	75.6%	0.001
Cisgender woman	225	37.0%	106	32.3%		45	7.7%	20	16.3%	
Transgender man/woman	52	8.6%	15	4.6%		19	3.2%	8	6.5%	
Not known/refused/missing	0	0.0%	7	2.1%		0	0.0%	2	1.6%	
**Sexual partner type** ^b^										
Main partners	68	11.2%	26	52.0%		154	26.2%	26	21.1%	0.92
Causal partners	11	1.8%	20	40.0%		399	68.0%	62	50.4%	
Sex work/exchange partners	1	0.2%	4	8.0%		28	4.8%	5	4.1%	
Not known/refused/missing	-- ^c^	-- ^c^	-- ^c^	-- ^c^		6	1.0%	30	24.4%	
**HIV status**										
HIV positive	107	17.6%	16	4.9%	<0.001	72	15.3%	4	3.3%	0.002
HIV negative	453	74.5%	173	52.7%		318	54.6%	79	64.2%	
Not known/refused/missing	48	7.9%	139	42.4%		197	33.5%	40	32.5%	
**Criminal justice involvement**										
Ever incarcerated	149	24.5%	71	21.7%	0.87	137	27.6%	22	17.9%	0.89
Never incarcerated	435	71.6%	213	64.9%		360	61.3%	60	48.8%	
Not known/refused/missing	24	4.0%	44	13.4%		90	15.3%	41	33.3%	
**Group sex**										
Ever had group sex	128	21.1%	40	12.2%	0.41	149	42.5%	15	12.2%	0.004
No previous group sex	361	59.4%	134	40.9%		202	29.5%	49	39.8%	
Not known/refused/missing	119	19.6%	154	47.0%		234	40.0%	59	48.0%	
**Sex drug use**										
Ever used drugs during sex	169	39.3%	0	0.0%	<0.00	229	53.4%	22	17.9%	0.005
Never used drugs during sex	261	60.7%	77	23.5%		200	46.5%	42	34.2%	
Not known/refused/missing	178	29.3%	251	76.5%		158	26.9%	59	48.0%	

^a^ All univariate analyses exclude network members for whom information was not known or for which participant’s refused to answer; ^b^ As it was possible for participant to identify sex partners who were also confidants, sexual partner types are reported for both networks (*n* = 130); ^c^ ‘Missing’ in this context may refer to unknown data or confidants who were not sexual partners, and have therefore been suppressed for these strata.

## Abbreviations

ART	antiretroviral treatment
CSV	comma separated value
HIV	human immunodeficiency virus
ERGMs	exponential random graph models
GIS	geographic information systems
GPS	global positioning system
LGBT	lesbian, gay, bisexual and transgender
MSM	men who have sex with men
N2	Neighborhoods and Networks
PrEP	pre-exposure prophylaxis
RDS	respondent-driven sampling
STIs	sexually transmitted infections

## Appendix A. Research and Coordination Sites

The N2 Study focused on three cities in the U.S.: Chicago, Illinois, Jackson, Mississippi, and New Orleans, Louisiana. The cohort’s management and implementation were locally coordinated by a research center in Chicago and community-based organizations operating in Jackson and New Orleans. The Spatial Epidemiology Lab at the New York University (NYU) School of Medicine (www.spatialepilab.org) serves as the central coordinating study and data center for the N2 Study. NYU’s Spatial Epidemiology Lab is a dynamic and active research group focused on connecting neighborhoods and health. By using innovative methods such as GPS technologies, the lab aims to relate neighborhood attributes to health-related behaviors and outcomes such as sexual health behaviors and HIV. The lab has an emphasis on intersectionality and health disparities among vulnerable populations, with a strong focus on sexual and gender minority populations, especially cisgender Black gay, bisexual and other men who have sex with men (MSM), and transgender women of color.

### Appendix A.1. Chicago Site: Chicago Center for HIV Elimination

The Chicago Center for HIV Elimination (https://hivelimination.uchicago.edu), which oversees the study site in Chicago, is a research and health service center with separate analytic and community facing units within the University of Chicago School of Medicine on Chicago’s South Side. The center was established with the goal of eliminating new HIV transmission events in the U.S. by 2041. The center hybridizes network science, next-generation testing and notification, integrated bio-behavioral prevention, and community mobilization to eliminate new transmission events in Chicago. Much of the center’s research focuses on HIV prevention and care among younger Black MSM and helps activate networks of community members to engage in health care. An estimated 3500 Black MSM have participated in several research and service programs at the center since 2008, which represents over half of young Black MSM in this community area [104]. All the research visits for participants in the Chicago, Illinois MSA are being completed at the Chicago Center for HIV Elimination’s off campus community space, known as ‘The Village’.

### Appendix A.2. Jackson Site: My Brother’s Keeper, Inc.

My Brother’s Keeper, Inc. (http://mbkinc.org), which coordinates the study’s implementation in the Jackson, Mississippi MSA, is a private, non-profit, 501(c)(3) organization with the mission of reducing health disparities by enhancing the health and well-being of minority and marginalized populations through leadership in public and community health practices, collaborations, and partnerships. The organization was established in 1999 as a nascent entity dedicated to the prevention and care of persons infected with HIV. In 2012, My Brother’s Keeper, Inc. opened the Open Arms Healthcare Center, the first community-based, primary care clinic with a particular focus on the health needs of LGBT youth and adults in Mississippi. Currently, Open Arms Healthcare Center is a standalone, community-based primary healthcare and research center that actively sees over 1800 patients and research participants each year. An estimated 40% of these patients/participants are Black MSM. All the research visits for participants in the Jackson, Mississippi MSA are completed at the Open Arms Healthcare Center, which is housed under the auspices of My Brother’s Keeper.

### Appendix A.3. New Orleans Site: Brotherhood, Inc.

The study’s implementation in the New Orleans MSA is managed by Brotherhood, Inc. (http://brotherhoodinc.org). Brotherhood, Inc. is a non-profit community-based organization that provides housing (transitional and low-income), health education, various trainings, and linkages to support services catering to vulnerable populations in New Orleans, such as HIV-positive individuals as well as Black MSM and Black transgender women through education, enlightenment, and empowerment. Brotherhood, Inc. partners with a diverse arsenal of organizations that include New Orleans Regional AIDS Planning Council, the Louisiana HIV Prevention Community Planning Group, the American Public Health Association, the American Red Cross, and the National Minority AIDS Council to provide wrap-around services to address client needs. Through robust initiatives, Brotherhood, Inc. strives to tackle the economic, social, and health disparities of underserved communities throughout New Orleans. They oversee many projects such as one known as ‘T.H.R.I.V.E. NOLA’, which addresses the behavioral, psychological and socioeconomic conditions that place vulnerable populations at higher risk for contracting HIV, and Trinity and Doll House, which provide social/housing services and a safe haven for persons living with HIV and transgender women, significantly reducing the social and economic barriers to attaining healthcare services and security.
